# RNA Viruses in Aquatic Unicellular Eukaryotes

**DOI:** 10.3390/v13030362

**Published:** 2021-02-25

**Authors:** Mohammadreza Sadeghi, Yuji Tomaru, Tero Ahola

**Affiliations:** 1 Department of Microbiology, Faculty of Agriculture and Forestry, University of Helsinki, 00014 Helsinki, Finland; 2 Environment and Fisheries Applied Techniques Research Department, Fisheries Technology Institute, Fisheries Research and Education Agency, Hatsukaichi, Hiroshima 739-0452, Japan; tomaruy@affrc.go.jp

**Keywords:** protist, +ssRNA viruses, *Marnaviridae*, RdRp, ecology, sequence diversity, viral species, metagenomics, virus isolation, protist viruses

## Abstract

Increasing sequence information indicates that RNA viruses constitute a major fraction of marine virus assemblages. However, only 12 RNA virus species have been described, infecting known host species of marine single-celled eukaryotes. Eight of these use diatoms as hosts, while four are resident in dinoflagellate, raphidophyte, thraustochytrid, or prasinophyte species. Most of these belong to the order *Picornavirales*, while two are divergent and fall into the families *Alvernaviridae* and *Reoviridae*. However, a very recent study has suggested that there is extraordinary diversity in aquatic RNA viromes, describing thousands of viruses, many of which likely use protist hosts. Thus, RNA viruses are expected to play a major ecological role for marine unicellular eukaryotic hosts. In this review, we describe in detail what has to date been discovered concerning viruses with RNA genomes that infect aquatic unicellular eukaryotes.

## 1. Introduction

Simple unicellular eukaryotes (protists) are supposed to have evolved over one billion years ago in the Proterozoic oceans [1,2]. Unicellular eukaryotes established a distinct life-type organization with several types of intracellular membranous organelles. Most are essentially aquatic or semi-aquatic microorganisms, highly diverse and numerous, exhibiting many variations in form and function, with cells ranging in size from <1 micron to several millimeters, including phototrophs, phagotrophs (i.e., predatory forms), mixotrophs (that are both photosynthetic and phagotrophic), and osmotrophs, and encompassing various parasites or parasitoids of larger organisms in addition to free-living forms [3,4]. Most are categorized as ‘protists’, though there is an uncertain demarcation between protists and unicellular forms of fungi [5].

Marine unicellular protists include a heterogeneous collection of phototrophic (photosynthetic, also known as phytoplankton) and heterotrophic (nonphotosynthetic, also known as bacterioplankton) organisms with a wide variety of cell sizes and belonging to almost all eukaryotic lineages [4,6]. Marine microbial communities are incredibly diverse, and in addition to the prokaryotes that represent the popular understanding of ‘microbe’, they include interconnected groups of viruses and unicellular eukaryotes [7,8]. Virus-like particles (VLPs) have been reported in eukaryotic algae since the early 1970s [9], however, most reports describe the microscopic observations of the viruses, and whenever the particles emerged, they were not further characterized in the laboratory to achieve better understanding of these viruses.

In the early 1980s, the new circumstances compelled a change, and many large and small DNA viruses were discovered with double-stranded genomes, ones that infect a cultivable, unicellular, eukaryotic-like variety of algal species such as a subset of green algae [10,11]. For the past two decades, much research has focused on the diversity of ocean DNA viruses, mostly phages with large DNA genomes [12,13,14,15]. Nonetheless, considerable effort has also gone into obtaining a better understanding of the eukaryote-infecting viruses in aquatic ecosystems, since viruses have an ecological impact and also play essential roles in the origin, evolution, and mortality of aquatic protists [16,17].

Researchers have also paid increasing attention to the diversity and significance of RNA viruses living in marine ecosystems. For the first time, a positive-sense, single-stranded RNA (+ssRNA) virus that infects the raphidophyte *Heterosigma akashiwo* was isolated in 2003; it was reported as an example of an RNA virus infecting a marine protist [18]. This paved the way for the discovery of several related ssRNA viruses (picorna-like) that infect microalgae [19,20,21,22,23]. With few exceptions, all of these viruses, according to newly approved taxonomy, are classified as members of the order *Picornavirales* [24,25].

In addition, evidence has been steadily accumulating that RNA viruses are important contributors to marine protist ecology [26,27,28,29,30,31,32]. Molecular surveys targeting the RNA-dependent RNA polymerase (RdRp) gene of picorna-like viruses have shown the presence of diverse picornavirus sequences in seawater [33,34]. The sea-water-sample metagenomic study of the RNA viral genome suggests that the picornaviruses are dominant marine RNA viruses, but other diverse RNA viruses, including double-stranded RNA (dsRNA), exist as well [35,36]. Interestingly, marine RNA phages have been infrequently found, and presumably the dominant RNA viruses infect the diverse and abundant marine single-cell eukaryotes, rather than infecting the prokaryotes [14].

Despite this progress, the aquatic RNA virus community remains largely uncharacterized. With the development of metagenomic technologies [37,38,39,40], a continuous flow of new unidentified virus sequences from eukaryotic organisms makes it clear that knowledge of the protist RNA virus dataset remains extremely limited, far behind that of animal, plant, or fungal species [35,41,42]. Recently, metagenomic analysis targeting RNA viruses from a single aquatic habitat in an estuary area in China discovered over 4000 distinct RNA viruses, which doubled the previously known set of RNA viruses [42]. This work found several previously unrecognized virus groups at the levels of class, order, and family, representing all the +RNA virus phyla (*Lenarviricota*, *Pisuviricota*, and *Kitrinoviricota*), but identified only six dsRNA viruses (*Duplornaviricota*) [42]. Although multiple virus groups were identified, phylogenetic analyses of the RdRp protein show that the newly discovered viruses belong to the previously designated phyla of RNA viruses [42,43,44,45,46].

Marine virus communities have been reviewed from various perspectives in the past [16,29,30,47,48]. We believe that the time is appropriate for a new review, for several reasons. Firstly, several ssRNA viruses have been discovered, mostly from diatoms, over the past 10 years, ones that are worthy of attention. Secondly, some diatom viruses are being taken into culture, and their features are now under analysis [49]. Adding data regarding these novel diatom viruses will further highlight the remarkable variety of algal viruses in nature. Thirdly, environmental analyses of marine microbial assemblages are gaining more attention; to study the considerable unexplored genetic diversity of RNA viruses in the ocean, it is therefore worthwhile to summarize what we currently know about specific viral–host relationships [50,51]. Additionally, no review has focused exclusively on RNA viruses and their protist hosts together. To this end, we have collected details on 12 viruses with an RNA genome that are thus far known to infect specific single-celled organisms.

## 2. Marine RNA Viruses

In the early 1990s, researchers found that marine viruses are directly pathogenic for various ocean organisms [52]. It has been well established that marine viruses are the most abundant biological entities in oceanic marine environments, reaching up to 10^8^ viruses mL^−1^. This has further stimulated marine virus research [53]. Rapid advances in metagenomics have subsequently yielded vast numbers of sequences from different types of aquatic ecosystems, providing us with access to an assemblage of viruses of diverse unicellular eukaryotes [35,41,42,44,45]. Metagenomic studies of aquatic environments suggest the possibility of a massive presence of viral genotypes in surface- and deep-water samples from different kinds of marine habitats [41,54]. Many RNA viruses in aquatic environments belong to genetically diverse known populations and are phylogenetically related to particular host species [30]. Their association with diseases and the mortality of marine mammals, fish, and crustaceans is evident [55,56,57]. They have also been implicated in both coral bleaching and in the die-off of economically valuable bivalves [58,59]. With metagenomic methods now involved in the field of virology, multicellular organisms infected with marine RNA viruses have been under the spotlight. Now this research has broadened to include RNA virus isolates infecting protists [30]. Although metagenomics is a good method for analyzing all viruses, it needs to be optimized in order to detect particles with an RNA genome; this led to underestimation of the importance of RNA viruses. The viruses’ hosts are not generally recognized by metagenomic analysis, but some of the RNA sequences extracted from aquatic environments are phylogenetically linked to viruses with known hosts [42,44].

## 3. Protist Virus Characterization

Viruses infecting single-celled eukaryotes have been studied using two distinct approaches, namely culture-based and non-culture-based (PCR, metagenomics). This review mainly deals with protist viruses discovered prior to 2020, by means of a culture-based approach. The success of culture-based approaches to virus isolation is often critically dependent on skill and experience, and among laboratories, strategies for the culture-based approach differ [21,22,32,45,60,61,62,63,64]. Although all of these techniques successfully isolate the virus from the protists, because no comparative research uses different methodologies, it is impossible to declare whether any one method is more efficient than another.

Turning to culture-independent approaches, these have increasingly served in analysis of the protist virome. For the comprehensive analysis of a specific group of viruses, amplicon-based metagenomics are crucial, but using this approach is still an issue and, what remains a challenge is to investigate the entire viral community. One of the main problems is related to the primer design strategy for virus detection. Degenerate PCR primers targeting conserved amino acids in the highly conserved RdRp were used, finding highly diverse picornavirus-like viral sequences in seawaters around the USA [33,34]. The large amount of genetic diversity between virus families makes it challenging to detect multiple virus families. Furthermore, primer design is also dependent on pre-existing knowledge of the viral genomes. In recent years, unbiased metagenomic approaches have achieved growing popularity and allow the analysis of the entire community of viruses. Additionally, metagenomics has the potential to detect even entirely novel viruses, because no previous knowledge of the assemblage is required [12,28,35,42]. More recently, a new protocol, one which uses a pair of degenerate PCR primers targeting replication protein regions of ssRNA and ssDNA viruses, suggests that the diverse RdRp-type viruses have some relationships with specific diatom host strains [51].

## 4. Taxonomy of Protist Viruses

The constant increase in the number of viruses, depositing genome sequence data from viruses in public archives, and advances in metagenomics, create more challenges in virus classification and initiate the opportunity to develop a new virus megataxonomy, one mostly based on metagenomic sequencing [42]. Many publications indicate that RNA viruses using RdRp are monophyletic with respect to the RdRp palm subdomain [65,66,67,68,69]. There exists no evidence for multiple origins of RNA viruses that have been isolated from eukaryotic and prokaryotic hosts, implying that they are most likely monophyletic and ancient. As such, they would qualify for an independent major taxon on their own in virus taxonomy, while also comprising a class of the Baltimore classification [46,65]. Thus far, the best-characterized group of protist viruses is the family *Marnaviridae* (Table 1). Among all of the protist virus isolates, two are divergent and fall in the families of *Alvernaviridae* and *Reoviridae* (Table 2) [24,25]. Metagenomic discovery will most likely increase the number of virus species known to infect related aquatic protists. Notably, one recent study discovered 854 new picorna-like viruses from one location in China, of which 653 fell into the *Marnaviridae* [42].

### 4.1. Picornaviridae in Protists

Isolation and identification of a picorna-like virus that causes lysis of the toxic, bloom-forming microalga *Heterosigma akashiwo* during the early steps of infection was the first evidence that RNA viruses are pathogens of marine phytoplankton [18]. Later, metagenomic sequencing from environmental samples established the enormous diversity in seawater viruses; the majority of them are classified in the order *Picornavirales*. The analysis of environmental picorna-like virus RdRp sequences mapped a distinct phylogenetic cluster of marine viruses genomes that probably describe primarily the virus infection of protists [26,30,33,34,35,70,71]. A metagenomic survey of marine RNA virus assemblages demonstrated that the most abundant reads among coastal RNA viruses belonged to the picorna-like viruses [35].

**Figure 1:**
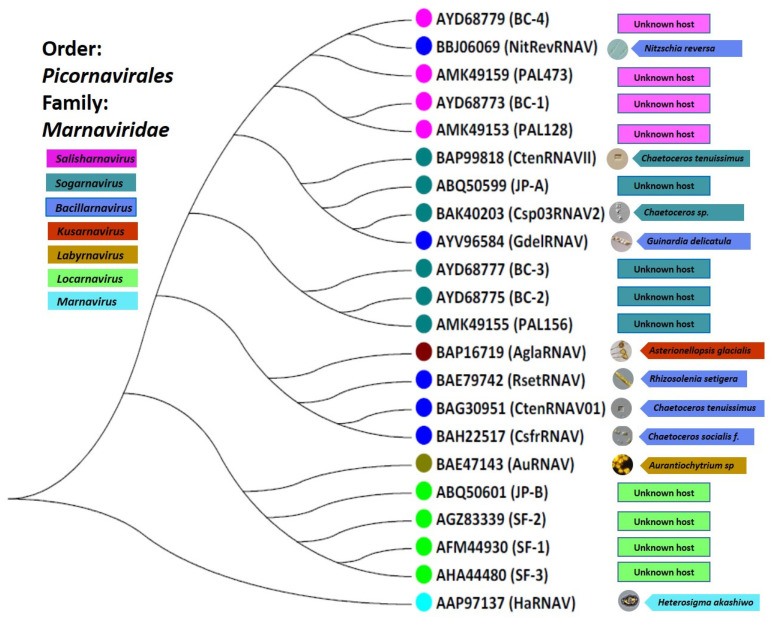
Maximum-likelihood phylogenetic analysis of the RdRp domain RNA sequences of the 22 aquatic RNA virus sequences belonging to the family *Marnaviridae*. The phylogenetic tree was constructed by the maximum likelihood (ML) method using MEGA 7.0 software. Illustration by authors.

The *Picornavirales* order unites small, non-enveloped viruses that infect eukaryotes and possess a number of prominent features. Members of this order are +ssRNA viruses with a genome length between 7.2 and 9.8 kb. They have an icosahedral virion (approximately 30 nm in size) that has a pseudo T = 3 architecture. All members of the order *Picornavirales* contain a Hel–Pro–Pol (Helicase, Protease and RdRp) replication block [72]. Members of the order *Picornavirales* are known to be able to infect a wide range of species, including vertebrates (birds, fish, amphibians, reptiles, and mammals), invertebrates (insects), microalgae, human beings, and plants [72]. Picornaviruses are associated with a mild, severe, and lethal range of human diseases such as aseptic meningitis, encephalitis, the common cold, febrile rash illnesses, conjunctivitis, herpangina, myositis and myocarditis, and hepatitis. Prior to the metagenomic discovery of 4500 distinct RNA viruses [42], we analyzed the conserved RdRp domains’ amino acid sequences of the 10 isolates and 12 metagenomically assembled genomes of +ssRNA viruses related to the order *Picornavirales* which are presented in Table 1. The sequence-based taxonomy for the order *Picornavirales* in connection with their protist hosts is presented in Figure 1. Currently, the order *Picornavirales* includes the families *Dicistroviridae*, *Iflaviridae*, *Marnaviridae*, *Picornaviridae*, *Polycipiviridae*, *Secoviridae Caliciviridae*, and *Solinviviridae*, [73,74]. Recently, all protist picorna-like viruses were joined to the family *Marnaviridae* which includes seven genera: *Labyrnavirus*, *Bacillarnavirus*, *Marnavirus*, *Locarnavirus*, *Kusarnavirus*, *Salisharnavirus*, and *Sogarnavirus* [28]. However, it is clear that this group requires a taxonomic reorganization based on new data [42].

**Table 1:** RNA viruses discussed in this review in the order *Picornavirales*.

	Protist Viruses in the Order *Picornavirales*							
Genus	Species/Virus	Virus Name Abbreviation	Genome		Host or Source		Country	References
			Size (nt)	ORFs ^1^				
*Marnavirus*	*Heterosigma akashiwo RNA virus*	HaRNAV	8587	One	Raphidophyte	*Heterosigma akashiwo*	Canada	Lang et al., 2004; Tai et al., 2003 [18,23]
*Labyrnavirus*	*Aurantiochytrium ssRNA virus*	AuRNAV01	9035	Two	Thraustochytrids	*Aurantiochytrium sp.*	Japan	Takao et al., 2006 [75]
*Locarnavirus*	Marine RNA virus JP-B	JP-B	8926	Two	Unknown	Coastal marine	Canada	Culley et al., 2007 [70]
	Marine RNA virus SF-2	SF-2	9321	Two	Unknown	Coastal wastewater	USA	Greninger and DeRisi, 2015 [27]
	Marine RNA virus SF-1	SF-1	8970	Two	Unknown	Coastal wastewater	USA	Greninger and DeRisi, 2015 [27]
	Marine RNA virus SF-3	SF-3	8648	One	Unknown	Coastal wastewater	USA	Greninger and DeRisi, 2015 [27]
*Kusarnavirus*	*Asterionellopsis glacialis RNA virus*	AglaRNAV	8842	Two	Diatom	*Asterionellopsis glacialis*	Japan	Tomaru et al., 2012 [63]
*Bacillarnavirus*	*Chaetoceros tenuissimus RNA virus 01*	CtenRNAV Type 1	9431	Two	Diatom	*Chaetoceros tenuissimus*	Japan	Shirai et al., 2008 [76]
	*Rhizosolenia setigera RNA virus*	RsetRNAV	8877	Two	Diatom	*Rhizosolenia setigera*	Japan	Nagasaki et al., 2004 [22]
	*Chaetoceros socialis f. radians RNA virus 01*	CsfrRNAV	9467	Two	Diatom	*Chaetoceros socialis f. radians*	Japan	Tomaru et al., 2009 [62]
	*Guinardia delicatula RNA virus*	GdelRNAV	9233	Two	Diatom	*Guinardia delicatula*	France	Arsenieff et al., 2018 [60]
	*Nitzschia reversa RNA virus*	NitRevRNAV	~9000	Two	Diatom	*Nitzschia reversa*	Japan	Toyoda et al., 2019 [77]
*Salisharnavirus*	Marine RNA virus BC-4	BC-4	8593	Two	Unknown	Coastal/oceanic marine	Canada	Vlok et al., 2019b [47]
	Marine RNA virus PAL473	PAL473	6360	Two	Unknown	Coastal marine	USA	Miranda et al., 2016 [71]
	Marine RNA virus BC-1	BC-1	8638	Two	Unknown	Coastal marine	Canada	Vlok et al., 2019b [47]
	Marine RNA virus PAL128	PAL128	8660	Two	Unknown	Coastal marine	USA	Miranda et al., 2016 [71]
*Sogarnavirus*	Marine RNA virus BC-2	BC-2	8843	Two	Unknown	Coastal marine	Canada	Vlok et al., 2019b [47]
	Marine RNA virus PAL156	PAL156	7897	Two	Unknown	Coastal marine	USA	Miranda et al., 2016 [71]
	Marine RNA virus BC-3	BC-3	8496	Two	Unknown	Coastal marine	Canada	Vlok et al., 2019b [47]
	Marine RNA virus JP-A	JP-A	9236	Two	Unknown	Coastal marine	Canada	Culley et al., 2007 [70]
	*Chaetoceros tenuissimus RNA virus type II*	CtenRNAV Type 2	9562	Two	Diatom	*Chaetoceros tenuissimus*	Japan	Kimura and Tomarua, 2015 [78]
	*Chaetoceros species RNA virus01*	Csp03RNAV	9417	Two	Diatom	*Chaetoceros sp.*	Japan	Tomaru et al., 2013 [64]

^1^ Open Reading Frame.

### 4.2. Alvernaviridae in Protists

Dinoflagellates are a group of unicellular protists that serve as natural hosts for the *Alvernaviridae* family. To date, only one virus of this family has been reported: Heterocapsa circularisquama RNA virus 01 (HcRNAV01) (Table 2) [24]. HcRNAV01 is the only characterized ssRNA virus to infect a dinoflagellate. This virus encodes a serine proteinase which is crucial for the viral replication cycle [24]. It has been shown to be linked to the serine proteinase gene distantly related to that of a member of the plant-infecting genus *Sobemovirus* [79]. HcRNAV01 genomes encode two major open reading frames and a stem-loop structure at the 3′-end [24]. The complete nucleotide (nt) sequence and the genomic organization of HcRNAV01 is distinct from those of members of the genus *Sobemovirus* and the families *Luteoviridae* and *Barnaviridae*. Because of these unique properties, HcRNAV01 was assigned to a new genus (*Dinornavirus*) and a new family (*Alvernaviridae*) [80]. Two strains of HcRNAV01 (HcRNAV01 strain 34 and strain 109) have their entire genomic RNA sequences available [24]. The new study by Wolf et al. (2020) found 239 viruses classified in solemo-, alverna, and barna-like virus groups in China [42].

### 4.3. Reoviridae in Protists

The *Reoviridae* is a large family of viruses with genomes containing 10, 11, or 12 segments of double-stranded RNA (dsRNA). Micromonas pusilla reovirus (MpRV) is the first ds RNA virus yet discovered in the family *Reoviridae* that infects a protist (the prasinophyte microalga *Micromonas pusilla*, a member of the Mamiellophyceae) (Table 2) [25]. Viruses of this family include a total of 75 virus species with a further ~30 tentative species reported to date [73]. They have been isolated from a wide range of animal species including both vertebrates and invertebrates (mammals, birds, reptiles, fish, crustaceans, insects, ticks, and other arachnids), plants, and fungi. Interestingly, the recent metagenomics study found only six dsRNA viruses, suggesting that they may be rather rare in protist hosts [42].

**Table 2:** RNA viruses discussed in this review in the families *Alvernaviridae* and *Reoviridae.*

**Protist Viruses in the Family *Alvernaviridae***								
**Genus**	**Species**	**Virus Name Abbreviation**	**Genome**		**Host or Source**		**Country**	**References**
			**Size (nt)**	**ORFs ^1^**				
*Dinornavirus*	*Heterocapsa circularisquama* *RNA virus 01*	HcRNAV	4400	Two	Dinoflagellate	*Heterocapsa circularisquama*	Japan	Nagasaki et al., 2005a; Tomaru et al., 2004 [21,24]
**Protist Viruses in Family *Reoviridae***								
**Genus**	**Species**	**Virus Name Abbreviation**	**Genome**		**Host or Source**		**Country**	**References**
			**Size (nt)**	**ORFs**				
*Mimoreovirus*	*Micromonas pusilla* *reovirus*	MpRNAV-01B	4400	11	Prasinophyceae	*Microalga Micromonas pusilla*	France	Brussaard et al., 2004 [25]

^1^ Open Reading Frame.

## 5. Host Specificity of Protist Viruses

Viral replication involves several steps including attachment to the host cell, penetration, uncoating, replication, assembly, and finally release [81]. To date, the attachment and entry mechanisms utilized by algal viruses to infect their hosts are almost unknown. Compatibility between host and virus has, however, been investigated, revealing that it is critical for successful infection [82]. Studies have demonstrated that marine RNA viruses infecting protists are mostly quite host-specific, with some variability [18,22,83,84,85]. Algal viruses have shown until now differing abilities to infect certain species of hosts. They are able to infect only a single host strain, several genetically different host strains, and hosts from different but closely related or distantly related species [81,86].

HcRNAV infects the bivalve-killing dinoflagellate *Heterocapsa circularisquama* [21]. Intraspecies host specificity has been reported for HcRNAV [87]. High-frequency nt substitutions of HcRNAV structural open reading frame (ORF) are predicted to be located on the surface of the virus particle, making this virus capable of binding to surface receptors of different hosts [87]. Host specificities of the viruses seem to be determined by their surface capsid viral proteins (VPs) [49]. The major capsid protein VP1 is important for receptor binding, because sequence variation is a response to host evolution [49]. The N-terminus of VP1 is hydrophobic in picornaviruses; one suggestion is that it is responsible for the attachment of virus particles to the endosomal membrane during cell entry [88]. Similarly, the N-terminus of VP1 in CtenRNAV-II is hydrophobic and one theory is that it shares the same functional role in the infection process with some other members of *Picornavirales* [49]. For the marine RNA virus BC-3, the VP1 domain is most likely important for binding to the cell-surface receptor; thus, long-term virus–host coevolution seems to have given rise to a surprising outcome that would affect selection on this domain [47,89].

## 6. RNA Viruses of Diatoms

Diatoms, a group of photosynthetic protists belonging to the division Heterokontophyta (also known as stramenopiles), represent a major group of phytoplankton in both marine and freshwater environments [90]. Marine diatoms have been extant over the last hundred million years and today are the most species-rich group of microalgae. According to conservative estimates, they comprise more than 100,000 species [91]. Diatoms are globally distributed, occurring from tropical and subtropical regions to polar ecosystems [92], and they may contribute more than 40% of total marine primary productivity [91]. These photosynthetic workhorses occur in waters worldwide, wherever they find adequate nutrients and sunlight. Their rigid cell wall (termed a frustule) composed of silica is one of diatoms’ distinguishing characteristics. Frustules consist of two nearly identical halves (thecae) very similar in appearance to a cell culture plate coupled with its lid, these enclosing the cell. The shape of the diatom frustule symmetry usually divides these diatoms into two main groups. Centric diatoms have the priority to be radially symmetric, whereas pennate diatoms usually have parallel striae that tend to be elongated, arranged perpendicular to the long axis. Diatom host–virus relationships may have been established in the very early stages of the biological evolution of the diatoms on the basis of apparently similar morphological features between centric and pennate diatom virus features [63]

Diatoms have been well known as key players in the marine carbon cycle for many years [93,94], but the existence of virus species that can infect diatoms has been described very rarely. The analysis of phytoplankton sediment by electron microscopy has sometimes shown the presence of VLPs in unidentified diatom cells from the Pacific Ocean [95], but, with no reported isolation of diatom viruses. The first diatom virus was reported in 2004, an ssRNA virus infecting *Rhizosolenia setigera* [22]. Thereafter, several *Chaetoceros* viruses have been successfully isolated. Thus far, at least 15 diatom viruses have been isolated and characterized using culture-based approaches. Silica frustules might be assumed to act as a potent barrier against viral infection. However, the pores in the frustule indicate a possible way that a virus can enter a cell. Since the frustule pores in *R. setigera* (ca. 80 nm in diameter) and the *C. salsugineum* setae are larger than their respective pathogens RsetRNAV (32 nm) and CsalDNAV (38 nm), this difference in size may provide the viruses with a route of infection [20,22]. If viral particles need to bypass the frustule pores as the first step towards facilitating the successful infection of the diatom, this would represent a major evolutionary pressure for diatom viruses to be small. However, even if some pores are large, the great majority of the frustule surface has pores no larger than 5 to 10 nm in diameter, limiting viral ingress [96]. In fact, viruses do infect diatoms, and it is evident that, in coastal waters, silicon limitation facilitates virus infection and diatom mortality [97]. In the following sections, we summarized some of the most important features of diatom viruses isolated to date.

### 6.1. Diatoms of the Genus Rhizosolenia

*Rhizosolenia setigera* is a centric diatom which has both sexual and asexual reproduction cycles and has many chloroplasts located throughout each cell [98]. Its cells are cylindrical, with conical valves narrowing into a long, straight, and needle-like spine. It produces resting spores, one completely different from those of the mother cell [98]. It also produces chemicals (monocyclic alkenes) that may cause mortality in marine organisms due to oxygen depletion during bloom decay. It is mainly coastal and estuarine, though occasionally found in open oceans. *R. setigera* is eurythermal (found in temperatures ranging from −2 to 30 °C) and euryhaline (found in salinity ranging from 1.5 to 37 PSU—practical salinity unit). It blooms in shallow water embayments in late spring and early fall [99].

The Rhizosolenia setigera RNA virus (RsetRNAV) is an RNA virus that infects *R. setigera.* It was previously designated by the abbreviation RsetRNAV (Rhizosolenia setigera RNA virus) [22]. In spring 2002, an unknown species of cryptophyta was dominant in Ariake Sound in western Japan. This virus was first isolated from surface water samples that were inoculated into the growing cultures of the 22 diatom strains including *R. setigera* [22]. A complete annotated sequence of the virus genome was reported four years later [100]. RsetRNAV has an icosahedral capsid structure 32 nm in diameter, and the genome is a linear +ssRNA with a polyA tail at the 3′ end [22,100]. Additional information for each virus, such as virus genome size and open reading frame (ORF), are presented in Table 1. The major structural proteins of RsetRNAV are 41.5, 41.0, and 29.5 kDa.

**Figure 2:**
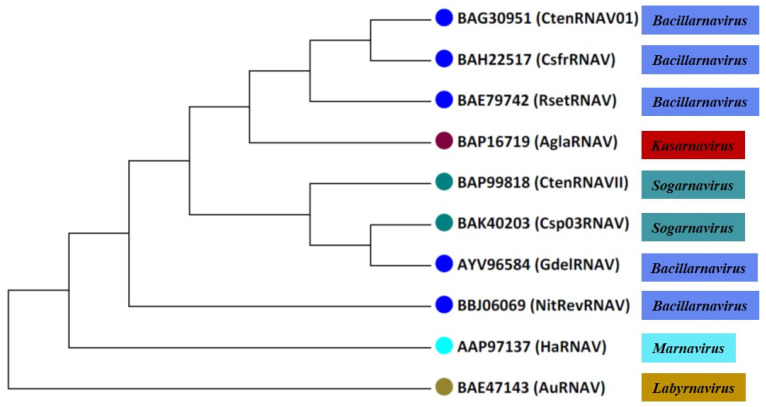
Comparative phylogenetic analysis of eight diatom viruses based on complete RNA sequences. This phylogenetic tree was constructed using the maximum likelihood (ML) method with the MEGA 7.0 software. RsetRNAV: Rhizosolenia setigera RNA virus; CsfrRNAV: Chaetoceros socialis f. radians RNA virus; CtenRNAV Type 1: Chaetoceros tenuissimus RNA virus Type I; CtenRNAV Type 2: Chaetoceros tenuissimus RNA virus Type II; Csp03RNAV: Chaetoceros sp. strain SS08-C03 RNA virus; GdelRNAV: Guinardia delicatula RNA virus; AglaRNAV: Asterionellopsis glacialis RNA virus; NitRevRNAV: Nitzschia reversa RNA virus; HaRNAV: Heterosigma akashiwo RNA virus; AuANAV: Aurantiochytrium ssRNA virus. Illustration by authors.

Virus particles accumulate in the cytoplasm of infected cells [22]. When viruses were inoculated in a given culture medium for each culture phase (stationary and exponential), the latent period (time between host infection and lysis) for RsetRNAV was 48 h and its burst size (fecundity, progeny produced per infected cell) was 1100 to 3000 infectious units per host cell [76,100]. The infection specificity of this virus, rather than being species specific, is strain specific, indicating that the virus sensitivities of diatoms, among host clones, are diverse. When the major structural proteins and genome of RsetRNAV were characterized [100], the phylogenetic tree thus constructed, based on RdRp amino acid sequences, shows that diatom-infecting ssRNA viruses (RsetRNAV01), along with other two-diatom viruses from the genus *Bacillarnavirus* (discussed in the following Section 6.2) that infect the hosts *Chaetoceros tenuissimus* and *Chaetoceros socialis f. radians,* form a monophyletic branch (Figure 1 and Figure 2).

### 6.2. Diatoms of the Genus Chaetoceros

The diatom genus *Chaetoceros* is one of the most abundant and diverse marine phytoplankton types in coastal and oceanic waters worldwide [91], with about 400 species reported [101]. To date, several RNA viruses have been isolated and characterized from this genus: Chaetoceros tenuissimus RNA virus (CtenRNAV type I), Chaetoceros socialis f. radians RNA virus (CsfrRNAV), Chaetoceros tenuissimus RNA virus (CtenRNAV type II), and Chaetoceros sp. strain SS08-C03 RNA virus (Csp03RNAV) [98].

**Figure 3:**
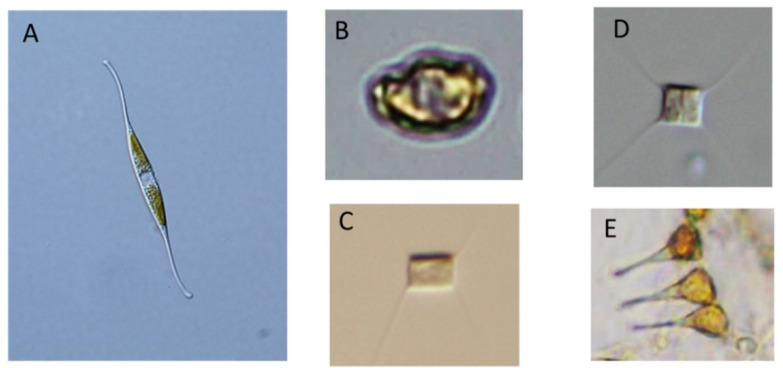
Microscopic images of the diatoms known to act as hosts for viruses: (**A**) *Nitzschia reversa*, host diatom for NitRevRNAV; (**B**) *Heterosigma akashiwo* host diatom for HaRNAV. (**C**,**D**) *Chaetoceros tenuissimus* host diatom for CtenRNAV II and CtenRNAV 01; (**E**) *Asterionellopsis glacialis* host diatom for AglaRNAV. Illustrations: (**A**) by Ken Toyoda from the Nippon Dental University, Tokyo, Japan; (**B**–**E**) by Yuji Tomaru from Fisheries Technology Institute, Fisheries Research and Education Agency, Hatsukaichi, Hiroshima, Japan.

CtenRNAV type I causes the lysis of the bloom-forming marine diatom *Chaetoceros tenuissimus Meunier* (Figure 3) [76]. CtenRNAV type I was first isolated from water samples of Ariake Sound in western Japan during June 2004 [76]. CtenRNAV type I has an icosahedral capsid structure which is 31 nm in diameter, and the genome is a linear +ssRNA and lacks a tail (Table 1) [76]. It has three major proteins (33.5, 31.5, and 30.0 kDa). The molecular weight of the major capsid proteins showed a size difference between RsetRNAV01 and CtenRNAV type I. The phylogenetic analysis of the RdRp sequence of CtenRNAV type I showed that this virus is closely related to RsetRNAV01, the type species of the genus *Bacillarnavirus* (Figure 1 and Figure 2). The RdRp amino acid sequence identity between RsetRNAV01 and CtenRNAV type I is 64.5%, a value well below the current species demarcation limit currently in use for most genera of the order *Picornavirales*. Another fundamental important difference between the two diatom-infecting virus species is their host organisms, differing at genus level.

CsfrRNAV is one more member of the genus *Bacillarnavirus* that was reported a decade ago [62]. CsfrRNAV causes the lysis of the bloom-forming species *Chaetoceros socialis Lauder f. radians (Schütt) Proschkina- Lavrenko*. CsfrRNAV was first isolated from environmental water samples of Hiroshima Bay in western Japan in April 2005. It is a very small polyhedral diatom virus of 22 nm in diameter, and the genome is a linear positive-stranded +ssRNA with a polyA tail at the 3′ end. It has three polypeptides of 32.0, 28.5, and 25.0 kDa. Virus assembly takes place in the host cell cytoplasm. The latent period and burst size of CsfrRNAV are <48 h and 66 infectious units per host cell [62]. The RdRp amino acid sequence identity between CsfrRNAV01 and RsetRNAV01 and between CsfrRNAV01 and CtenRNAV type I are 61.2 and 46.0%. The most important difference among the three diatom-infecting virus species is in their host organisms differing at the genus level: i.e., RsetRNAV01, CtenRNAV type I, and CsfrRNAV01 are, respectively, infectious to *Rhizosolenia setigera, Chaetoceros tenuissimus*, and *Chaetoceros socialis f. radians* [22,62,76].

CtenRNAV type II was isolated from sediment from Hiroshima Bay, Japan [78]. Its physiological and morphological characteristics are similar to those of the previously isolated CtenRNAV type I [76]. CtenRNAV type II has an icosahedral capsid structure of 35 nm in diameter, and its genome is a linear +ssRNA and lacks a tail (Table 1) [78]. It has three major proteins (32.2, 29.0, and 26.1 kDa). However, the amino acid sequences of the structural proteins CtenRNAV type II are clearly distinct from type I virus, with an identity of 27.6% [78]. This virus shows lytic activities to several diatom species within the genus *Chaetoceros*. Thus, CtenRNAV type-II infects multiple species [78]. Four ancestral structural traits were observable in the conformation of the VP1 EF-loop, the conformation of the VP1 CD-loop, the VP2 N-terminal domain swap, and the putative autoproteolytic motifs in VP1 and VP3. This finding was possible when the capsid structure of CtenRNAV-II was revealed by the structural determination of a virus protein by cryo-electron microscopy and compared with previously determined capsid protein structures of other viruses belonging to the same order infecting invertebrates, vertebrates, and plants [49].

Csp03RNAV, a member of the genus *Sogarnavirus* that causes the lysis of the marine planktonic diatom *Chaetoceros sp. strain SS08-C03*, was isolated from surface waters of the Yatsushiro Sea, Japan [64]. This virion is icosahedral and 32 nm in diameter, and its assembly takes place in the cytoplasm of the host cells. The latent period was estimated to be <48 h. The Csp03RNAV genome of 9417 bases encodes two ORFs, one of which codes for putative replication-related proteins and the other for putative structural proteins (Table 1). Csp03RNAV expressed three major polypeptides at 42.0, 34.0, and 28.0 kDa [64]. The monophyly of Csp03RNAV and the other known diatom-infecting ssRNA viruses (genus *Bacillarnavirus*), Rhizosolenia setigera RNA virus, Chaetoceros socialis f. radians RNA virus, and Chaetoceros tenuissimus RNA virus is supported by phylogenetic analysis based on the amino acid sequence of the conserved RdRp domain [64].

### 6.3. Diatom of the Genus Asterionella

*Asterionella* is a genus of pennate diatoms identified as freshwater diatoms [102]. They are frequently found in star-shaped colonies (consisting up to 20 cells, but often eight) of individuals [103]. The species *Asterionella glacialis* was first reported from the Indian sector of the Antarctic Ocean. It is an important contributor to global diatom phytoplankton [102]. A century later, this diatom was placed in a new genus to accommodate its marine habitat and the structure of the colony-linking apparatus, among other characters that differed from those of *Asterionella*. *Asterionellopsis glacialis* was the designated type species and is one of the few species currently assigned to this genus. The life histories of these diatoms are not well known, but involve characters demonstrated to be evolutionarily informative in other higher pennates [102].

*Asterionellopsis glacialis* (Figure 3)was isolated from the surface waters of Hiroshima Bay, Japan, and its infectious virus Asterionellopsis glacialis RNA virus (AglaRNAV) from sediments (0–1 cm depth) of Ago Bay, Japan [63]. The AglaRNAV has a linear +ssRNA genome of approximately 9.5 kb (Table 1); and its particle is 31 nm in diameter and accumulates in the host cytoplasm [63]. AglaRNAV has four major proteins. This virus belongs to the *Kusarnavirus* genus in the family *Marnaviridae.* This is the first isolation and preliminary characterization of pennate diatom viruses that infects *Asterionellopsis glacialis* [63].

### 6.4. Diatom of the Genus Guinardia

The diatom genus *Guinardia* is characterized as a major contributor to micro-phytoplankton assemblages along the Atlantic coasts, the North Sea, and the western Irish Sea [98]. The bloom-forming species *Guinardia delicatula* is one of the abundant diatom species in the German Bight [104,105,106]. Several groups of eukaryotic parasites are described as causing the mortality of this diatom. Its cells form fairly straight chains and are bilaterally symmetrical. The external process is thin and short, and is narrow, tube-shaped, and oblique to the pervalvar axis. The external process fits into a depression on the adjacent valve. Girdle segments are composed of open bands with poroid areolae, and are not prominent [98].

The Guinardia delicatula RNA virus (GdelRNAV) was isolated as the first virus that infects *G. delicatula* from a location in western England in the time of the late summer bloom decline of *G. delicatula* [60]. These lytic viruses replicate in the host cytoplasm and are small particles of 35–38 nm in diameter. GdelRNAV has a genome of ∼9 kb, including two ORFs encoding for replication and structural polyproteins (Table 1). It has five major proteins: 38.6, 33.9, 29.8, 27, and 6.8 kDa. GdelRNAV is specific to several strains of *G. delicatula* [60]. Based on the RdRp gene marker, GdelRNAV was placed in the genus *Bacillarnavirus* [60]; however, reclassification could be needed (Figure 1 and Figure 2).

### 6.5. Diatoms of the Genus Nitzschia

*Nitzschia* is a pennate diatom common in marine ecosystems [98]. Chilly waters are their preferred site. Furthermore, *Nitzschia* often occurs as one of the dominant diatoms in the Arctic and Antarctic polar sea ice [98]. Several species are reported for this diatom, a toxicologically known species which produces domoic acid, a known neurotoxin responsible for human amnesic shellfish poisoning (ASP) [107].

Nitzschia reversa RNA virus (NitRevRNAV) was isolated from surface seawaters of Sagami Bay, Japan, with the pennate diatom *Nitzschia reversa* (Figure 3) [77]. The host specificity of this virus was revealed by inoculating various diatom species, such as *Nitzschia spp.*, *Cylindrotheca spp.*, *Chaetoceros spp.*, *Skeletonema spp.*, and *Achnanthes spp.*, with virus suspensions. The virus was lytic to its original host, *N. reversa* strain KT30, but not to other microalgal species tested. These results indicated the high species-specific infection of this virus, which is a general feature of microalgal viruses [22,62]. The size of these virus particles, in diameter, was 30 nm, as shown by transmission electron microscopy (TEM) after negative staining. The NitRevRNAV genome is a linear +ssRNA with a poly(A) tail (Table 1) [77]. It has four major polypeptides at 36, 32, 30, and 28 kDa. Phylogenetic analysis of amino acid sequences of RdRp placed NitRevRNAV as a member of the genus *Bacillarnavirus* [77]; however, the reclassification could be required (Figure 1 and Figure 2).

## 7. Viruses of the Family Raphidophyceae

The Raphidophyceae are a small group of flagellated protists that inhabit diverse aquatic habitats [108]. To date, ten genera have been distinguished from marine, brackish, and freshwater: *Gonyostomum*, *Merotricha*, and *Vacuolaria* are three representatives for freshwater; seven other genera: *Chattonella*, *Chlorinimonas*, *Fibrocapsa*, *Haramonas*, *Heterosigma*, *Psammamonas*, and *Viridilobus* have brackish species [109]. The Raphidophyceae are photosynthetic and belong to a phylum of unicellular wall-less heterokonts. They have two flagella in the apex of the cell that contain tubular mastigonemes [110]. Some of the marine species contain the marine carotenoid fucoxanthin, whereas freshwater species do not contain this pigment. Marine raphidophytes are extensively recognized as ichthyotoxic organisms, and a relationship has emerged between finfish mortality and several species like *Chattonella spp*., *Fibrocapsa japonica*, and *Heterosigma akashiwo*. To understand the mechanisms of bloom formation, it is important to collect information on the raphidophyte life cycle, on cyst formation, and on vertical migratory behavior [111]. Viruses assigned to the genus *Heterosigma* infect the harmful bloom-forming raphidophyte, *Heterosigma akashiwo* (a member of the family Raphidophyceae), a marine alga with a world-wide distribution. *Heterosigma akashiwo* is a harmful bloom-forming alga living in temperate coastal waters. Since factors affecting its occurrence and bloom formation have remained elusive, the bloom’s timing and the severity of this eukaryotic algae are both still unknown [112].

Heterosigma akashiwo RNA virus (HaRNAV) belongs to the genus *Marnavirus*. HaRNAV infects several strains of *Heterosigma akashiwo* from coastal British Columbian waters (Figure 3). This was the first reported ssRNA virus that caused the lysis of a phytoplankton species [18]. The HaRNAV complete genome was sequenced a year after discovery, and its genome sequence predicted a single ORF encoding a polyprotein that contains conserved picorna-like protein domains, with putative nonstructural protein domains present in the N-terminus and the structural proteins in the C-terminus of the polyprotein [23]. The virus genome is 8587 nt in length, plus a poly(A) tail (Table 1). The genome sequence determined contains one large ORF on the positive strand that is 7743 bases long and is predicted to encode a protein of 2581 amino acid residues. The 5′and 3′ untranslated regions (UTRs) are 483 and 361 nt long, respectively, accounting for a total of 9.8% of the genome. The secondary structure near the 5′ end is likely to be functionally important in this virus for the replication of the RNA, as seen in other picorna-like viruses. HaRNAV particles contain five proteins: ones of 33.9, 29.0, 26.1, 24.6, and 24.0 kDa. It has an icosahedral capsid structure of 25 nm in diameter [18]. Secondary structures close to the start of the polyprotein are likely functionally important as part of an internal ribosome entry site (IRES) for the translation of the polyprotein, as in other picorna-like viruses. There exists a notable pyrimidine-rich stretch of sequence wherein 22 of 29 bases are pyrimidines; this ends at eight bases upstream of the predicted start codon of the large ORF. Such sequences are conserved in picorna-like viruses and are important as part of the IRES [23].

The infectivity of HaRNAV was tested against 15 strains of *Heterosigma akashiwo* isolated from Japanese waters, the northeast Pacific, and the northwest Atlantic [18]. HaRNAV caused lysis of three strains from the northeast Pacific and two strains from Japan, but none from the northwest Atlantic. The discovery of HaRNAV emphasizes the diversity of *Heterosigma akashiwo* viral pathogens and more importantly, sheds light on algal–virus pathogens and the complexity of virus–host interactions in the environmental protist [18]. HaRNAV was the first picornalike virus described that infects a protist, and it is the only virus characterized in the genus *Marnavirus,* to date [18].

## 8. Viruses of the Family Thraustochytriaceae

Species of the genus *Thraustochytrium,* in particular, have become of increasing interest to biotechnology research [113]. The Thraustochytriaceae, together with several other families, form the order Thraustochytrida, which, together with the orders Labyrinthulida and Amphitremida, belong to the class Labyrinthulomycota [114]. The Labyrinthulomycota belong among marine, saprotrophic, fungus-like, unicellular organisms. They lack a plasmid and are described by (their) bothrosome, by atubulocristate mitochondria, and by Golgi-derived scales. [115].

The genus *Schizochytrium* had been previously accepted in the class Labyrinthulomycetes, family Thraustochytriaceae, within the kingdom Chromista. It has been reconsidered with a more precise classification of the *Schizochytrium* by supplementing its morphological, physiological, and molecular phylogenetic characteristic data [116]. SssRNAV is infectious to the marine fungoid protist *Schizochytrium* sp. NIBH N1-27 [19,75]. As a result of taxonomic rearrangement, all SssRNAV-sensitive host strains were placed in the genus *Aurantiochytrium,* thereby establishing a new species for this virus. This virus received the name Aurantiochytrium single-stranded RNA virus 01(AuRNAV01).

AuRNAV01, the first RNA virus infecting marine fungoid protists, was isolated from the coastal waters of Kobe Harbor, Japan, in July 2000 [19]. All AuRNAV particles showed the same icosahedral capsid protein and were approximately 25 nm in diameter. The assembly of virus capsids takes place in the cytoplasm of the host cells. The viral RNA genome is 9018 nt in length (excluding the 3′ poly A tail) (Table 1). The virus genome contains two long ORFs, which are separated by an intergenic region of 92 nt. The 5′ ORF 1 is preceded by an un-translated leader sequence of 554 nt. The downstream large ORF 2 and an additional ORF 3 overlap by 431 nt; ORF 3 is followed by an un-translated region of 70 nt (excluding the 3′ poly A tail). AuRNAV01 has three major proteins (37, 34, and 32 kDa), and two minor proteins (80 and 18 kDa) [19].The three ORFs (ORF1, ORF2, and ORF3) encode three different proteins: putative replication proteins (ORF1), capsid proteins (ORF2), and a protein of unknown function (ORF3). The results obtained by northern blot analysis suggest that AuRNAV01 synthesizes sub-genomic RNAs to translate ORF2 and ORF3 [75]. Virus particles can form crystalline arrays and move random assemblies within the cytoplasm of host cells. The lytic cycle was estimated at < 8 h, and the burst size at 5.8×10^3^‒6.4×10^4^ infectious units per host cell [19]. The putative replication proteins and capsid protein sequences have revealed notably high similarity to the diatom-infecting viruses RsetRNAV01, CtenRNAV01, and CsfrRNAV01, as well as to the HaRNAV-SOG263 from the family *Marnaviridae* [62,75]. However, some properties of this virus are clearly distinct from the other viruses infecting protists. The phylogenetic analysis of the RdRp nt and amino acid sequence has established that AuRNAV01 forms a separate branch distinct from that of related viruses, with its closest related viruses being the three diatom-infecting viruses [62,75].

## 9. Viruses of Dinoflagellates

Dinoflagellates are a unique group of unicellular organisms classified among the harmful phytoplankton species. They are well known because of their having high morphological biodiversity and species richness, and several adaptation strategies to survive in various ecological niches. [117]. They are autotrophs, mixotrophs, osmotrophs, symbionts, and parasites. A high number of dinoflagellate species have been revealed to be mixotrophic, and their highly aggressive feeding behavior enables them to adapt and develop their nutrient absorption, helping them to survive under undesirable conditions which are difficult for strict autotrophs to survive in [2,118,119]. One of the important features making dinoflagellates crucial to marine ecosystems is the ecotoxicological effect. Dinoflagellates are fundamentally significant, since they show the highest representation among toxic phytoplankton, with 99 species, in contrast to the number of diatom species (29), Raphidophyceans (4), and Cyanobacteria [119].

*Heterocapsa circularisquama Horiguchi* is a harmful bloom-forming dinoflagellate that specifically infects and kills bivalves; there exist multiple viruses known to infect *Heterocapsa circularisquama* [21,22,120,121]. Heterocapsa circularisquama RNA virus (HcRNAV) is thus far the only virus in the family *Alvernaviridae* and genus *Dinornavirus*. HcRNAV, a ssRNA virus specifically infecting *Heterocapsa circularisquama*, which was maintained in culture and isolated from the coastal waters of Japan. HcRNAV strains were grouped into two types, depending upon intra-species host-range analysis. These two types indicated complementary strain-specific infectivity. Typical strains of each type (HcRNAV34 and HcRNAV109) have been characterized. Both virus strains have capsids with icosahedral symmetry and a size of 30 nm in diameter, and their genome is ssRNA which is approximately 4.4 kb in size (Table 2). HcRNAV strains have one major polypeptide with a molecular weight of 38 kDa [21]. Thus, in morphology and nucleic-acid type, HcRNAV is distinct from HcDNAV, the previously reported large double-stranded DNA virus infecting *Heterocapsa circularisquama*. Virus-particle assembly takes place in the cytoplasm of the host cells within 24 h post-infection, with crystalline arrays or unordered aggregations of virus particles observable. The burst size has been estimated at 3.4 × 10^3^ to 2.1 × 10^4^ infectious particles cell^−1^, and the latent period at 24 to 48 h [21,22,120,121].

The finding of RNA viruses infecting microalgae such as HaRNAV and HcRNAV emphasizes the diversity of algicidal viral pathogens [85]. One study indicates that during the maximum of one *Heterocapsa circularisquama* bloom, a substantial 88% of cells contained VLPs similar to the size of the corresponding HcRNAV [22]. Furthermore, a clear association has been observable in HcRNAV abundance in water and sediments, with the population of host cells in the water column [32], suggesting that HcRNAV can play a major role in *Heterocapsa circularisquama* population dynamics. Another finding is that, in those ecosystems with potentially important ecological consequences, where the dinoflagellate is the host, differing responses to HaRNAV infection have been evident. Some *Heterocapsa circularisquama* strains display a “delayed-lysis” property which allows them in some way to appear resistant to infection, with no culture lysis detectable; these cultures, however, actually produce as much progeny virus as emerges in a completely lysed culture [83].

An important characteristic of the *Heterocapsa circularisquama* vs. the HcRNAV relationship is that the host culture shows disintegration due to HcRNAV inoculations, but some percentage of the cells can survive through virus infection. The survivor cells re-grow under HcRNAV pressure. Intracellular viral RNA replication was assumed to be interrupted in the virus-resistant cells [122].

## 10. Viruses of the Family Prasinophyceae

Prasinophytes (Chlorophyta) constitute a group of unicellular algae at the base of the green algal lineage. They include several marine photosynthetic picoeukaryote species described from cultured isolates [123,124,125]. Several studies have demonstrated the importance of eukaryotic picoplankton (cell size, 0.2 to 3 μm) in terms of biomass and productivity in the euphotic zone of oceanic oligotrophic waters, as well as in coastal waters [126,127,128]. At present, nine prasinophyte clades are recognized, most corresponding to existing orders [129]. Within marine picoplankton, *Micromonas pusilla* (the only species described in the genus *Micromonas*) has been identified as a major component of the picoplanktonic community in several oceanic and coastal regions [130].

Micromonas pusilla RNA virus (MpRNAV-01B) or *Micromonas pusilla* reovirus (MpRV) is the first double-stranded RNA virus in the family *Reoviridae* and subfamily *Sedoreovirinae* genus *Mimoreovirus* that infects the photosynthetic marine picoflagellate *M. pusilla* [25]. An 11 segment of the dsRNA genome has been identified for this virus (Table 2), one clearly distinct from that of the other viruses of the family *Reoviridae* with 11-segmented genomes, namely the *rotaviruses* and *aquareoviruses*. It is noteworthy that the segment 1 of MpRV with 5792 bp has the longest open reading frame of any of the thus-far characterized reoviruses [25]. MpRNAV has a particle size of 65–80 nm and contains five major proteins (120, 95, 67, 53, and 32 kDa). The RNA-dependent RNA polymerases have been found to be encoded by Segment 2 of MpRV. A partial genome identity (21%) occurs between MpRV and the Human rotavirus C from the family of *Reoviridae* with 11-segmented dsRNA virus within the enzyme core region of RdRp. The terminal sequences of MpRV are distinct from all those of sequenced viruses of the family *Reoviridae*. Phylogenetic analysis based on the RdRp sequences showed that MpRV cannot be grouped with any of the previously characterized genera of viruses and takes its position within another phylogenetic group. Therefore, MpRV was introduced as a member of a new and distinct genus designated *Mimoreovirus* [25]. MpRNAV-01B shows the ability to remain stable during freezing and thawing, and it is resistant to chloroform, ether, nonionic detergents, and to chelating and reducing agents. The virus is inactive at temperatures above 35 °C and is resistant to ionic detergents, ethanol, acetone, and acidic conditions (pH 2–5) [25].

## 11. Conclusions

Marine microorganisms comprise a major portion of the living biomass on the globe, thereby driving ecological cycling and the flows of energy. While only comprising a small part of the total marine biomass, viruses dominate in abundance and genetic variability [15,131,132]. The data presented in this review indicate that several important eukaryotic algal types such as diatoms, dinoflagellates, Raphidophytes, Thraustochytrids, and Prasinophyceae are exposed to viral attacks, but the specifying of host-virus pairs remains a major challenge.

Screening each of the isolated viruses against other potential hosts than the original can answer important questions regarding protist viruses. No definitive relationship is yet known between diatoms and their viruses in situ; however, some authors believe that their ecological interactions are observable in nature, based on field surveys and physiological studies [61,76]. It is of great interest to examine the mechanisms supporting the strain-specific infectivity of these viruses, and the host–receptor and virus binding–site relationships [49]. Intraspecies host specificity of the dinoflagellate virus HcRNAV has thus far been determined to be at the early steps of infection [87].

The virus community can change the abundance and habitant of its hosts, meaning that diatoms have potential importance in controlling the quantity (biomass) and quality (clonal composition) of diatom populations in the natural environment [133]. Innovation in enumerating each host species and the viruses it harbors (such as by real-time PCR or metagenomics) should pave the way for the better understanding of their interactions. The field of marine viral ecology is in its early stages, due to the lack of data regarding the ways in which diverse viruses interact with their hosts under varying environmental conditions.

It is striking that such a wide variety of evolutionarily distant protist hosts are infected by the relatively closely related viruses of the family *Marnaviridae* (Table 1, Figure 1), and that the studies of protist host–virus relationships have thus far been dominated by this group of viruses. Comparative studies of these viruses with each other and with further members of the *Picornavirales* should reveal the secrets of their success. Although, *Marnaviridae* is definitely a major group, it only represents ~15% of the new marine viruses discovered in the recent metagenomic study [42], many of which likely use protists as hosts. Thus, the immaturity of the protist-virus study field is easy to recognize. Given the ancient branching of protists from the lineages leading to multicellular eukaryotes, we believe that studies of protist viruses and virus–host interactions at ecological, cell biological and molecular levels will illuminate the evolutionary history of all RNA viruses [42,134].

## References

[1] Javaux E.J., Knoll A.H., Walter M.R. (2001). Morphological and ecological complexity in early eukaryotic ecosystems. Nature.

[2] Adachi M. (1998). Molecular classification and identification of toxic dinoflagellate, Alexandrium. Nippon Suisan Gakk.

[3] Park J.S., Simpson A.G. (2015). Diversity of Heterotrophic Protists from Extremely Hypersaline Habitats. Protist.

[4] Adl S.M., Bass D., Lane C.E., Lukes J., Schoch C.L., Smirnov A., Agatha S., Berney C., Brown M.W., Burki F. (2019). Revisions to the Classification, Nomenclature, and Diversity of Eukaryotes. J. Eukaryot. Microbiol..

[5] James T.Y., Pelin A., Bonen L., Ahrendt S., Sain D., Corradi N., Stajich J.E. (2013). Shared signatures of parasitism and phylogenomics unite Cryptomycota and microsporidia. Curr. Biol..

[6] Burki F., Roger A.J., Brown M.W., Simpson A.G.B. (2020). The New Tree of Eukaryotes. Trends Ecol. Evol..

[7] Breitbart M., Salamon P., Andresen B., Mahaffy J.M., Segall A.M., Mead D., Azam F., Rohwer F. (2002). Genomic analysis of uncultured marine viral communities. Proc. Natl. Acad. Sci. USA.

[8] Djamali E., Nulton J.D., Turner P.J., Rohwer F., Salamon P. (2012). Heat output by marine microbial and viral communities. J. Non-Equil. Thermody.

[9] Lee R.E. (1971). Systemic viral material in the cells of the freshwater red alga Sirodotia tenuissima (Holden) skuja. J. Cell Sci..

[10] Van Etten J.L., Lane L.C., Meints R.H. (1991). Viruses and viruslike particles of eukaryotic algae. Microbiol. Rev..

[11] Van Etten J.L., Meints R.H., Burbank D.E., Kuczmarski D., Cuppels D.A., Lane L.C. (1981). Isolation and characterization of a virus from the intracellular green alga symbiotic with Hydra viridis. Virology.

[12] Kristensen D.M., Mushegian A.R., Dolja V.V., Koonin E.V. (2010). New dimensions of the virus world discovered through metagenomics. Trends Microbiol..

[13] Edwards R.A., Rohwer F. (2005). Viral metagenomics. Nat. Rev. Microbiol..

[14] Weinbauer M.G. (2004). Ecology of prokaryotic viruses. FEMS Microbiol. Rev..

[15] Porter A.F., Shi M., Eden J.S., Zhang Y.Z., Holmes E.C. (2019). Diversity and Evolution of Novel Invertebrate DNA Viruses Revealed by Meta-Transcriptomics. Viruses.

[16] Suttle C.A. (2007). Marine viruses—Major players in the global ecosystem. Nat. Rev. Microbiol..

[17] Rohwer F., Thurber R.V. (2009). Viruses manipulate the marine environment. Nature.

[18] Tai V., Lawrence J.E., Lang A.S., Chan A.M., Culley A.I., Suttle C.A. (2003). Characterization of HaRNAV, a single-stranded RNA virus causing lysis of Heterosigma akashiwo (Raphidophyceae). J. Phycol..

[19] Takao Y., Nagasaki K., Mise K., Okuno T., Honda D. (2005). Isolation and characterization of a novel single-stranded RNA virus infectious to a marine fungoid protist, Schizochytrium sp (Thraustochytriaceae, labyrinthulea). Appl. Environ. Microbiol..

[20] Nagasaki K., Tomaru Y., Takao Y., Nishida K., Shirai Y., Suzuki H., Nagumo T. (2005). Previously unknown virus infects marine diatom. Appl. Environ. Microbiol..

[21] Tomaru Y., Katanozaka N., Nishida K., Shirai Y., Tarutani K., Yamaguchi M., Nagasaki K. (2004). Isolation and characterization of two distinct types of HcRNAV, a single-stranded RNA virus infecting the bivalve-killing microalga Heterocapsa circularisquama. Aquat. Microb. Ecol..

[22] Nagasaki K., Tomaru Y., Katanozaka N., Shirai Y., Nishida K., Itakura S., Yamaguchi M. (2004). Isolation and characterization of a novel single-stranded RNA virus infecting the bloom-forming diatom Rhizosolenia setigera. Appl. Environ. Microbiol..

[23] Lang A.S., Culley A.I., Suttle C.A. (2004). Genome sequence and characterization of a virus (HaRNAV) related to picorna-like viruses that infects the marine toxic bloom-forming alga Heterosigma akashiwo. Virology.

[24] Nagasaki K., Shirai Y., Takao Y., Mizumoto H., Nishida K., Tomaru Y. (2005). Comparison of genome sequences of single-stranded RNA viruses infecting the bivalve-killing dinoflagellate Heterocapsa circularisquama. Appl. Environ. Microbiol..

[25] Brussaard C.P., Noordeloos A.A., Sandaa R.A., Heldal M., Bratbak G. (2004). Discovery of a dsRNA virus infecting the marine photosynthetic protist Micromonas pusilla. Virology.

[26] Gustavsen J.A., Winget D.M., Tian X., Suttle C.A. (2014). High temporal and spatial diversity in marine RNA viruses implies that they have an important role in mortality and structuring plankton communities. Front. Microbiol..

[27] Greninger A.L., DeRisi J.L. (2015). Draft Genome Sequences of Marine RNA Viruses SF-1, SF-2, and SF-3 Recovered from San Francisco Wastewater. Genome Announc..

[28] Vlok M., Lang A.S., Suttle C.A. (2019). Application of a sequence-based taxonomic classification method to uncultured and unclassified marine single-stranded RNA viruses in the order Picornavirales. Virus Evol..

[29] Steward G.F., Culley A.I., Mueller J.A., Wood-Charlson E.M., Belcaid M., Poisson G. (2013). Are we missing half of the viruses in the ocean?. ISME J..

[30] Lang A.S., Rise M.L., Culley A.I., Steward G.F. (2009). RNA viruses in the sea. FEMS Microbiol. Rev..

[31] Tomaru Y., Fujii N., Oda S., Toyoda K., Nagasaki K. (2011). Dynamics of diatom viruses on the western coast of Japan. Aquat. Microb. Ecol..

[32] Tomaru Y., Hata N., Masuda T., Tsuji M., Igata K., Masuda Y., Yamatogi T., Sakaguchi M., Nagasaki K. (2007). Ecological dynamics of the bivalve-killing dinoflagellate Heterocapsa circularisquama and its infectious viruses in different locations of western Japan. Environ. Microbiol..

[33] Culley A.I., Lang A.S., Suttle C.A. (2003). High diversity of unknown picorna-like viruses in the sea. Nature.

[34] Culley A.I., Steward G.F. (2007). New genera of RNA viruses in subtropical seawater, inferred from polymerase gene sequences. Appl. Environ. Microbiol..

[35] Culley A.I., Lang A.S., Suttle C.A. (2006). Metagenomic analysis of coastal RNA virus communities. Science.

[36] Urayama S., Takaki Y., Nishi S., Yoshida-Takashima Y., Deguchi S., Takai K., Nunoura T. (2018). Unveiling the RNA virosphere associated with marine microorganisms. Mol. Ecol. Resour..

[37] Sadeghi M., Popov V., Guzman H., Phan T.G., Vasilakis N., Tesh R., Delwart E. (2017). Genomes of viral isolates derived from different mosquitos species. Virus Res..

[38] Sadeghi M., Kapusinszky B., Yugo D.M., Phan T.G., Deng X.T., Kanevsky I., Opriessnig T., Woolums A.R., Hurley D.J., Meng X.J. (2017). Virome of US bovine calf serum. Biologicals.

[39] Sadeghi M., Altan E., Deng X.T., Barker C.M., Fang Y., Coffey L.L., Delwart E. (2018). Virome of > 12 thousand Culex mosquitoes from throughout California. Virology.

[40] Delwart E.L. (2007). Viral metagenomics. Rev. Med. Virol..

[41] Bench S.R., Hanson T.E., Williamson K.E., Ghosh D., Radosovich M., Wang K., Wommack K.E. (2007). Metagenomic characterization of Chesapeake Bay virioplankton. Appl. Environ. Microbiol..

[42] Wolf Y.I., Silas S., Wang Y., Wu S., Bocek M., Kazlauskas D., Krupovic M., Fire A., Dolja V.V., Koonin E.V. (2020). Doubling of the known set of RNA viruses by metagenomic analysis of an aquatic virome. Nat. Microbiol..

[43] Zhang Y.Z., Chen Y.M., Wang W., Qin X.C., Holmes E.C. (2019). Expanding the RNA virosphere by unbiased metagenomics. Annu. Virol..

[44] Dolja V.V., Koonin E.V. (2018). Metagenomics reshapes the concepts of RNA virus evolution by revealing extensive horizontal virus transfer. Virus Res..

[45] Culley A.I., Mueller J.A., Belcaid M., Wood-Charlson E.M., Poisson G., Steward G.F. (2014). The characterization of RNA viruses in tropical seawater using targeted PCR and metagenomics. mBio.

[46] Koonin E.V., Dolja V.V., Krupovic M., Varsani A., Wolf Y.I., Yutin N., Zerbini F.M., Kuhn J.H. (2020). Global Organization and Proposed Megataxonomy of the Virus World. Microbiol. Mol. Biol. Rev..

[47] Vlok M., Lang A.S., Suttle C.A. (2019). Marine RNA Virus Quasispecies Are Distributed throughout the Oceans. mSphere.

[48] Yau S., Seth-Pasricha M. (2019). Viruses of Polar Aquatic Environments. Viruses.

[49] Munke A., Kimura K., Tomaru Y., Okamoto K. (2020). Capsid Structure of a Marine Algal Virus of the Order Picornavirales. J. Virol..

[50] Greninger A.L. (2018). A decade of RNA virus metagenomics is (not) enough. Virus Res..

[51] Tomaru Y., Kimura K. (2020). Novel Protocol for Estimating Viruses Specifically Infecting the Marine Planktonic Diatoms. Diversity.

[52] Suttle C.A., Chan A.M., Cottrell M.T. (1991). Use of ultrafiltration to isolate viruses from seawater which are pathogens of marine phytoplankton. Appl. Environ. Microbiol..

[53] Bergh O., Borsheim K.Y., Bratbak G., Heldal M. (1989). High abundance of viruses found in aquatic environments. Nature.

[54] Angly F.E., Felts B., Breitbart M., Salamon P., Edwards R.A., Carlson C., Chan A.M., Haynes M., Kelley S., Liu H. (2006). The marine viromes of four oceanic regions. PLoS Biol..

[55] Bonami J.R., Zhang S. (2011). Viral diseases in commercially exploited crabs: A review. J. Invertebr. Pathol..

[56] Mortensen H.F., Heuer O.E., Lorenzen N., Otte L., Olesen N.J. (1999). Isolation of viral haemorrhagic septicaemia virus (VHSV) from wild marine fish species in the Baltic Sea, Kattegat, Skagerrak and the North Sea. Virus Res..

[57] Jensen T., van de Bildt M., Dietz H.H., Andersen T.H., Hammer A.S., Kuiken T., Osterhaus A. (2002). Another phocine distemper outbreak in Europe. Science.

[58] Renault T., Novoa B. (2004). Viruses infecting bivalve molluscs. Aquat. Living Resour..

[59] Levin R.A., Voolstra C.R., Weynberg K.D., van Oppen M.J. (2017). Evidence for a role of viruses in the thermal sensitivity of coral photosymbionts. ISME J..

[60] Arsenieff L., Simon N., Rigaut-Jalabert F., Le Gall F., Chaffron S., Corre E., Com E., Bigeard E., Baudoux A.C. (2018). First Viruses Infecting the Marine Diatom Guinardia delicatula. Front. Microbiol..

[61] Tomaru Y., Shirai Y., Suzuki H., Nagumo T., Nagasaki K. (2008). Isolation and characterization of a new single-stranded DNA virus infecting the cosmopolitan marine diatom Chaetoceros dehilis. Aquat. Microb. Ecol..

[62] Tomaru Y., Takao Y., Suzuki H., Nagumo T., Nagasaki K. (2009). Isolation and characterization of a single-stranded RNA virus infecting the bloom-forming diatom Chaetoceros socialis. Appl. Environ. Microbiol..

[63] Tomaru Y., Toyoda K., Kimura K., Hata N., Yoshida M., Nagasaki K. (2012). First evidence for the existence of pennate diatom viruses. ISME J..

[64] Tomaru Y., Toyoda K., Kimura K., Takao Y., Sakurada K., Nakayama N., Nagasaki K. (2013). Isolation and characterization of a single-stranded RNA virus that infects the marine planktonic diatom Chaetoceros sp (SS08-C03). Phycol. Res..

[65] Kuhn J.H., Wolf Y.I., Krupovic M., Zhang Y.Z., Maes P., Dolja V.V., Koonin E.V. (2019). Classify viruses—The gain is worth the pain. Nature.

[66] Poch O., Blumberg B.M., Bougueleret L., Tordo N. (1990). Sequence Comparison of 5 Polymerases (L-Proteins) of Unsegmented Negative-Strand Rna Viruses—Theoretical Assignment of Functional Domains. J. Gen. Virol..

[67] Vieth S., Torda A.E., Asper M., Schmitz H., Gunther S. (2004). Sequence analysis of L RNA of Lassa virus. Virology.

[68] Jacome R., Becerra A., de Leon S.P., Lazcano A. (2015). Structural Analysis of Monomeric RNA-Dependent Polymerases: Evolutionary and Therapeutic Implications. PLoS ONE.

[69] Shi M., Lin X.D., Tian J.H., Chen L.J., Chen X., Li C.X., Qin X.C., Li J., Cao J.P., Eden J.S. (2016). Redefining the invertebrate RNA virosphere. Nature.

[70] Culley A.I., Lang A.S., Suttle C.A. (2007). The complete genomes of three viruses assembled from shotgun libraries of marine RNA virus communities. Virol. J..

[71] Miranda J.A., Culley A.I., Schvarcz C.R., Steward G.F. (2016). RNA viruses as major contributors to Antarctic virioplankton. Environ. Microbiol..

[72] Le Gall O., Christian P., Fauquet C.M., King A.M., Knowles N.J., Nakashima N., Stanway G., Gorbalenya A.E. (2008). Picornavirales, a proposed order of positive-sense single-stranded RNA viruses with a pseudo-T = 3 virion architecture. Arch. Virol..

[73] Walker P.J., Siddell S.G., Lefkowitz E.J., Mushegian A.R., Dempsey D.M., Dutilh B.E., Harrach B., Harrison R.L., Hendrickson R.C., Junglen S. (2019). Changes to virus taxonomy and the International Code of Virus Classification and Nomenclature ratified by the International Committee on Taxonomy of Viruses (2019). Arch. Virol..

[74] Wolf Y.I., Kazlauskas D., Iranzo J., Lucia-Sanz A., Kuhn J.H., Krupovic M., Dolja V.V., Koonin E.V. (2018). Origins and Evolution of the Global RNA Virome. mBio.

[75] Takao Y., Mise K., Nagasaki K., Okuno T., Honda D. (2006). Complete nucleotide sequence and genome organization of a single-stranded RNA virus infecting the marine fungoid protist Schizochytrium sp.. J. Gen. Virol..

[76] Shirai Y., Tomaru Y., Takao Y., Suzuki H., Nagumo T., Nagasaki K. (2008). Isolation and characterization of a single-stranded RNA virus infecting the marine planktonic diatom Chaetoceros tenuissimus Meunier. Appl. Environ. Microbiol..

[77] Toyoda K., Kimura K., Osada K., Williams D.M., Adachi T., Yamada K., Tomaru Y. (2019). Novel marine diatom ssRNA virus NitRevRNAV infecting Nitzschia reversa. Plant Ecol. Evol..

[78] Kimura K., Tomarua Y. (2015). Discovery of Two Novel Viruses Expands the Diversity of Single-Stranded DNA and Single-Stranded RNA Viruses Infecting a Cosmopolitan Marine Diatom. Appl. Environ. Microb..

[79] Somera M., Sarmiento C., Truve E. (2015). Overview on Sobemoviruses and a Proposal for the Creation of the Family Sobemoviridae. Viruses.

[80] Adams M.J., Carstens E.B. (2012). Ratification vote on taxonomic proposals to the International Committee on Taxonomy of Viruses (2012). Arch. Virol..

[81] Short S.M. (2012). The ecology of viruses that infect eukaryotic algae. Environ. Microbiol..

[82] Tarutani K., Nagasaki K., Yamaguchi M. (2000). Viral impacts on total abundance and clonal composition of the harmful bloom-forming phytoplankton Heterosigma akashiwo. Appl. Environ. Microbiol..

[83] Mizumoto H., Tomaru Y., Takao Y., Shirai Y., Nagasaki K. (2008). Diverse responses of the bivalve-killing dinoflagellate Heterocapsa circularisquama to infection by a single-stranded RNA virus. Appl. Environ. Microbiol..

[84] Kimura K., Tomaru Y. (2017). Effects of temperature and salinity on diatom cell lysis by DNA and RNA viruses. Aquat. Microb. Ecol..

[85] Tomaru Y., Toyoda K., Kensuke K.K. (2015). Marine diatom viruses and their hosts: Resistance mechanisms and population dynamics. Perspect. Phycol..

[86] Tarutani K., Nagasaki K., Yamaguchi M. (2006). Virus adsorption process determines virus susceptibility in Heterosigma akashiwo (Raphidophyceae). Aquat. Microb. Ecol..

[87] Mizumoto H., Tomaru Y., Takao Y., Shirai Y., Nagasaki K. (2007). Intraspecies host specificity of a single-stranded RNA virus infecting a marine photosynthetic protist is determined at the early steps of infection. J. Virol..

[88] Fricks C.E., Hogle J.M. (1990). Cell-induced conformational change in poliovirus: Externalization of the amino terminus of VP1 is responsible for liposome binding. J. Virol..

[89] Rossmann M.G., He Y.N., Kuhn R.J. (2002). Picornavirus-receptor interactions. Trends Microbiol..

[90] Pierella Karlusich J.J., Ibarbalz F.M., Bowler C. (2020). Phytoplankton in the Tara Ocean. Ann. Rev. Mar. Sci..

[91] Malviya S., Scalco E., Audic S., Vincenta F., Veluchamy A., Poulain J., Wincker P., Iudicone D., de Vargas C., Bittner L. (2016). Insights into global diatom distribution and diversity in the world’s ocean. Proc. Natl. Acad. Sci. USA.

[92] Follows M.J., Dutkiewicz S., Grant S., Chisholm S.W. (2007). Emergent biogeography of microbial communities in a model ocean. Science.

[93] Smetacek V. (1999). Diatoms and the ocean carbon cycle. Protist.

[94] Smetacek V. (2018). Seeing is Believing: Diatoms and the Ocean Carbon Cycle Revisited. Protist.

[95] Proctor L.M., Fuhrman J.A. (1991). Roles of Viral-Infection in Organic Particle-Flux. Mar. Ecol. Prog. Ser..

[96] Raven J.A., Waite A.M. (2004). The evolution of silicification in diatoms: Inescapable sinking and sinking as escape?. New Phytol..

[97] Kranzler C.F., Krause J.W., Brzezinski M.A., Edwards B.R., Biggs W.P., Maniscalco M., McCrow J.P., Van Mooy B.A.S., Bidle K.D., Allen A.E. (2019). Silicon limitation facilitates virus infection and mortality of marine diatoms. Nat. Microbiol..

[98] Finlay B.J. (2006). The Kingdom Protista: The Dazzling World of Living Cells By Jeremy Pickett-Heaps and Julianne Pickett-Heaps. Protist.

[99] Hernandez-Becerril D.U., Del Castillo M.E.M. (1996). The marine planktonic diatom Rhizosolenia robusta (Bacillariophyta): Morphological studies support its transfer to a new genus, Calyptrella gen. nov. Phycologia.

[100] Shirai Y., Takao Y., Mizumoto H., Tomaru Y., Honda D., Magasaki K. (2006). Genomic and phylogenetic analysis of a single-stranded RNA virus infecting Rhizosolenia setigera (Stramenopiles: Bacillariophyceae). J. Mar. Biol. Assoc. UK.

[101] Tas S., Hernandez-Becerril D.U. (2017). Diversity and distribution of the planktonic diatom genus Chaetoceros (Bacillariophyceae) in the Golden Horn Estuary (Sea of Marmara). Diatom Res..

[102] Kaczmarska I., Mather L., Luddington I.A., Muise F., Ehrman J.M. (2014). Cryptic diversity in a cosmopolitan diatom known as Asterionellopsis glacialis (Fragilariaceae): Implications for ecology, biogeography, and taxonomy. Am. J. Bot..

[103] Kojadinovic-Sirinelli M., Villain A., Puppo C., Fon Sing S., Prioretti L., Hubert P., Gregori G., Zhang Y., Sassi J.F., Claverie J.M. (2018). Exploring the microbiome of the “star” freshwater diatom Asterionella formosa in a laboratory context. Environ. Microbiol..

[104] Gowen R.J., McCullough G., Kleppel G.S., Houchin L., Elliott P. (1999). Are copepods important grazers of the spring phytoplankton bloom in the western Irish Sea?. J. Plankton Res..

[105] Wiltshire K.H., Kraberg A., Bartsch I., Boersma M., Franke H.D., Freund J., Gebuhr C., Gerdts G., Stockmann K., Wichels A. (2010). Helgoland Roads, North Sea: 45 Years of Change. Estuar. Coast..

[106] Guilloux L., Rigaut-Jalabert F., Jouenne F., Ristori S., Viprey M., Not F., Vaulot D., Simon N. (2013). An annotated checklist of Marine Phytoplankton taxa at the SOMLIT-Astan time series off Roscoff (Western English Channel, France): Data collected from 2000 to 2010. Cah. Biol. Mar..

[107] Amzil Z., Fresnel J., Le Gal D., Billard C. (2001). Domoic acid accumulation in French shellfish in relation to toxic species of Pseudo-nitzschia multiseries and P. pseudodelicatissima. Toxicon.

[108] Vesk M., Moestrup O. (1987). The Flagellar Root-System in Heterosigma-Akashiwo (Raphidophyceae). Protoplasma.

[109] Yamaguchi H., Nakayama T., Murakami A., Inouye I. (2010). Phylogeny and taxonomy of the Raphidophyceae (Heterokontophyta) and Chlorinimonas sublosa gen. et sp. nov., a new marine sand-dwelling raphidophyte. J. Plant Res..

[110] Imai I., Yamaguchi M. (2012). Life cycle, physiology, ecology and red tide occurrences of the fish-killing raphidophyte Chattonella. Harmful Algae.

[111] Hiroishi S., Okada H., Imai I., Yoshida T. (2005). High toxicity of the novel bloom-forming species Chattonella ovata (Raphidophyceae) to cultured fish. Harmful Algae.

[112] Nagasaki K., Tarutani K., Yamaguchi M. (1999). Growth characteristics of Heterosigma akashiwo virus and its possible use as a microbiological agent for red tide control. Appl. Environ. Microbiol..

[113] Lee Chang K.J., Nichols C.M., Blackburn S.I., Dunstan G.A., Koutoulis A., Nichols P.D. (2014). Comparison of Thraustochytrids Aurantiochytrium sp., Schizochytrium sp., Thraustochytrium sp., and Ulkenia sp. for production of biodiesel, long-chain omega-3 oils, and exopolysaccharide. Mar Biotechnol (NY).

[114] Pan J., Del Campo J., Keeling P.J. (2017). Reference Tree and Environmental Sequence Diversity of Labyrinthulomycetes. J. Eukaryot. Microbiol..

[115] Adl S.M., Simpson A.G., Lane C.E., Lukes J., Bass D., Bowser S.S., Brown M.W., Burki F., Dunthorn M., Hampl V. (2012). The revised classification of eukaryotes. J. Eukaryot. Microbiol..

[116] Marchan L.F., Chang K.J.L., Nichols P.D., Mitchell W.J., Polglase J.L., Gutierrez T. (2018). Taxonomy, ecology and biotechnological applications of thraustochytrids: A review. Biotechnol. Adv..

[117] Gomez F. (2012). A quantitative review of the lifestyle, habitat and trophic diversity of dinoflagellates (Dinoflagellata, Alveolata). Syst. Biodivers..

[118] Sournia A. (1984). Classification and Nomenclature of Various Marine Dinoflagellates (Dinophyceae). Phycologia.

[119] Hoppenrath M. (2017). Dinoflagellate taxonomy—A review and proposal of a revised classification. Mar. Biodivers..

[120] Nagasaki K., Tomaru Y., Shirai Y., Takao Y., Mizumoto H. (2006). Dinoflagellate-infecting viruses. J. Mar. Biol. Assoc. UK.

[121] Tarutani K., Nagasaki K., Itakura S., Yamaguchi M. (2001). Isolation of a virus infecting the novel shellfish-killing dinoflagellate Heterocapsa circularisquama. Aquat. Microb. Ecol..

[122] Tomaru Y., Mizumoto H., Nagasaki K. (2009). Virus resistance in the toxic bloom-forming dinoflagellate Heterocapsa circularisquama to single-stranded RNA virus infection. Environ. Microbiol..

[123] Lemieux C., Otis C., Turmel M. (2014). Six newly sequenced chloroplast genomes from prasinophyte green algae provide insights into the relationships among prasinophyte lineages and the diversity of streamlined genome architecture in picoplanktonic species. BMC Genom..

[124] Kubiszyn A.M., Svensen C. (2018). First record of a rare species, Polyasterias problematica (Prasinophyceae), in Balsfjord, northern Norway. Bot. Mar..

[125] Sluiman H.J., Gartner G. (1990). Taxonomic Studies on the Genus Pleurastrum (Pleurastrales, Chlorophyta). I. The Type Species, Pleurastrum-Insigne, Rediscovered and Isolated from Soil. Phycologia.

[126] Garrido J.L., Rodriguez F., Zapata M. (2009). Occurrence of Loroxanthin, Loroxanthin Decenoate, and Loroxanthin Dodecenoate in Tetraselmis Species (Prasinophyceae, Chlorophyta)(1). J. Phycol..

[127] Jouenne F., Eikrem W., Le Gall F., Marie D., Johnsen G., Vaulot D. (2011). Prasinoderma singularis sp. nov. (Prasinophyceae, Chlorophyta), a solitary coccoid Prasinophyte from the South-East Pacific Ocean. Protist.

[128] Not F., Latasa M., Marie D., Cariou T., Vaulot D., Simon N. (2004). A single species, Micromonas pusilla (Prasinophyceae), dominates the eukaryotic picoplankton in the Western English Channel. Appl. Environ. Microbiol..

[129] Guillou L., Eikrem W., Chretiennot-Dinet M.J., Le Gall F., Massana R., Romari K., Pedros-Alio C., Vaulot D. (2004). Diversity of picoplanktonic prasinophytes assessed by direct nuclear SSU rDNA sequencing of environmental samples and novel isolates retrieved from oceanic and coastal marine ecosystems. Protist.

[130] Thomsen H.A., Buck K.R. (1998). Nanoflagellates of the central California waters: Taxonomy, biogeography and abundance of primitive, green flagellates (Pedinophyceae, Prasinophyceae). Deep-Sea Res. Pt Ii.

[131] Filippini M., Middelboe M. (2007). Viral abundance and genome size distribution in the sediment and water column of marine and freshwater ecosystems. FEMS Microbiol. Ecol..

[132] Breitbart M., Felts B., Kelley S., Mahaffy J.M., Nulton J., Salamon P., Rohwer F. (2004). Diversity and population structure of a near-shore marine-sediment viral community. Proc. Biol. Sci..

[133] Nagasaki K., Tomaru Y., Nakanishi K., Hata N., Katanozaka N., Yamaguchi M. (2004). Dynamics of Heterocapsa circularisquama (Dinophyceae) and its viruses in Ago Bay, Japan. Aquat. Microb. Ecol..

[134] Ahola T. (2019). New Phylogenetic Grouping of Positive-Sense RNA Viruses Is Concordant with Replication Complex Morphology. mBio.

